# Impact of chronic inflammatory airway disease on stroke severity and long-term survival after ischemic stroke - a retrospective analysis

**DOI:** 10.1186/s12883-015-0414-1

**Published:** 2015-09-08

**Authors:** Karl Georg Haeusler, Juliane Herm, Maria Konieczny, Ulrike Grittner, Mitja Lainscak, Matthias Endres, Wolfram Doehner

**Affiliations:** Department of Neurology, Charité, Universitätsmedizin Berlin, Berlin, Germany; Center for Stroke Research Berlin, Charité, Berlin, Germany; Department of Biostatistics and Clinical Epidemiology, Charité, Berlin, Germany; Departments of Cardiology and Research and Education, General Hospital Celje, Celje, Slovenia; Excellence Cluster NeuroCure, Charité, Universitätsmedizin Berlin, Berlin, Germany; German Center for Neurodegenerative Diseases (DZNE), Partner Site, Berlin, Germany; German Center for Cardiovascular Diseases (DZHK), Partner Site, Berlin, Germany; Department of Cardiology, Charité Universitätsmedizin Berlin, Campus Virchow, Berlin, Germany

## Abstract

**Background:**

Chronic inflammatory airway disease (CIAD) has emerged as independent risk factor for cardiovascular mortality and ischemic stroke but the impact of co-existing CIAD in patients with ischemic stroke is less clear.

**Methods:**

We retrospectively analyzed 1013 patients with acute ischemic stroke who were consecutively admitted to the Department of Neurology, Charité - Universitätsmedizin Berlin, Germany within one year. Mean follow-up was 80 months (IQR 32–85 months). Using multivariable regression models we analyzed the impact of CIAD (defined as chronic obstructive pulmonary disease or asthma bronchiale) on stroke severity and outcome.

**Results:**

Co-existing CIAD was evident in 7.1 % (*n* = 72) of all patients with acute ischemic stroke. Baseline characteristics of stroke patients with CIAD did not differ significantly from ischemic stroke patients without CIAD. Age (OR 1.17 [95 % CI 1.03-1.37] per decade), atrial fibrillation (OR 3.43 [95 % CI 2.47-4.78]) and coronary artery disease (OR 1.51 [95 % CI 1.07–2.14]) but not a history of CIAD (*p* = 0.30) were associated with severe stroke (NIHSS≥11) on hospital admission. Age (HR 1.70 [95 % CI 1.53-1.87] per decade), peripheral artery disease (HR 1.91 [95 % CI 1.35-2.7]), stroke severity at hospital admission (NIHSS per point HR 1.08 [95 % CI 1.06-1.10]), and history of CIAD (HR 1.43 [95 % CI 1.02-2.00]) were independently associated with mortality during long-term follow-up. However, CIAD was not significantly associated with short-term mortality after stroke.

**Conclusion:**

Co-existing CIAD showed no significant association with stroke severity at hospital admission and early mortality after ischemic stroke. CIAD was negatively associated with long-term survival after ischemic stroke.

## Background

Chronic inflammatory airway disease (CIAD) and ischemic stroke are both primarily affecting the elderly, and their prevalence is expected to rise in the near future. Even today, CIAD as well as ischemic stroke are leading causes of morbidity and mortality worldwide [1, 2]. In addition to the risk profile that CIAD and ischemic stroke have in common, available data indicate that CIAD itself increases the odds of having a stroke about 1.1- to 3.8-fold [3–8]. Various interrelated mechanisms may contribute to an increased stroke risk in CIAD-patients, i.e. chronic infection promoting large-artery atherosclerosis, hypoxia-induced systemic oxidative stress, hypercoagulability, endothelial dysfunction, increased thrombocyte aggregation, and atrial fibrillation [9–11]. The CIAD related mortality is likely underestimated because of the difficulties to identify the cause of death [12]. The severity of CIAD, advanced age and smoking may have an impact on the stroke risk of CIAD-patients [4, 13, 14].

The interaction of CIAD and stroke outcome is less well understood. A history of stroke was related to an increased in-hospital mortality in patients with exacerbated CIAD in a recent cross-sectional multicenter study [15]. In addition, stroke-associated death was inversely co-related to the forced expiratory volume within a prospective general population study [16]. Furthermore, CIAD is an independent risk factor for pneumonia after stroke [17], and, in turn, pneumonia is a major cause of death in stroke patients [18]. There is paucity, however, of epidemiological data regarding the frequency and impact of CIAD on acute stroke severity and long-term outcome after stroke.

The aims of this post-hoc study were: (I) to analyze the frequency of CIAD in patients with acute ischemic stroke; (II) to identify whether a history of CIAD is a relevant risk factor for the severity of acute ischemic stroke; (III) to investigate the impact of co-existing CIAD on survival after acute ischemic stroke.

## Methods

### Study design and study population

The retrospective study was conducted at the Department of Neurology, Charité - Universitätsmedizin Berlin and approved by the Charité Ethics Committee (EA1/186/07), waiving the need for obtaining informed consent. After defining the hypothesis of CIAD as an impact factor on outcome after ischemic stroke, medical records of all patients with acute ischemic stroke or TIA admitted to the three university hospitals of the Charité, Berlin, Germany, between 1st January and 31st December 2004 were analyzed retrospectively. Stroke or TIA patients were identified by using relevant ICD-10 discharge diagnoses (I61.x; I63.x; G45.x). All patients with ischemic stroke were included in the primary analysis. Additional analysis was performed combining patients with ischemic stroke and TIA. The following information was assessed from medical records: demographic details, medical history, antithrombotic medication and cardiovascular risk factors (e.g. atrial fibrillation, congestive heart failure, CIAD, hypertension, diabetes mellitus, previous stroke or TIA, intracerebral hemorrhage or non-stroke vascular events). Coexisting CIAD was defined as chronic obstructive pulmonary disease or asthma bronchiale and each diagnosis was identified from the medical records. Furthermore, in one patient presence of anti-obstructive medication (fluticasone and salmeterol) was taken into account. Stroke severity was assessed on admission according to the National Institutes of Health Stroke Scale (NIHSS) score [19].

Stroke severity was defined as “mild to moderate stroke” (NIHSS <11 points) and “severe stroke” (NIHSS ≥11 points) [20]. Functional status of patients on hospital admission was further assessed by the modified Rankin Scale (mRS) [21]. All patients were prospectively followed-up on survival status for up to 8 years after the index stroke. Survival status was obtained from the German central civil registration office. All patients without available survival status (because of an invalid address or registration) were excluded from survival analysis. All-cause mortality during follow-up was defined as the primary endpoint. “Short-term” mortality was defined as death ≤ 90 days after the index stroke, while “long-term” mortality was defined as death >90 days after stroke. We do not have detailed information on cause of death after hospital discharge.

### Statistical analysis

For categorical variables, absolute and relative frequencies were reported. In the case of continuous variables with normal distribution, the mean and standard deviation was reported. If continuous variables were non-normally distributed, the median and limits of interquartile range were reported. Distribution of variables was tested for normality using the Kolmogorov-Smirnov test. The *χ*^2^-test was used to test differences in proportions for dichotomous variables between independent groups. A linear trend test was used to analyze ordinal data. The Mann–Whitney-*U*-test was applied to analyze not normally distributed variables and multivariable logistic regression was applied as appropriate. Odds ratios (OR) with 95 % confidence intervals (CI) are reported for characteristics associated with stroke severity according to the NIHSS score or survival, respectively. Stepwise multivariable analysis was performed including all parameters with significant association to outcome according to bivariate analyses. Survival analyses were performed at 30 days, 90 days, and for the entire follow-up period. Cox proportional hazards analysis and log-rank test (Mantel Cox) were used for survival analyses. Hazard ratios (HR) and 95 % confidence intervals (CI) are presented. Multivariable analysis was performed with all parameters that showed significant associations in bivariate analyses using the enter method to ensure CIAD was included in the model. The survival function was estimated by the Kaplan-Meier method (product-limit estimator). A p-value of <0.05 was considered to be significant. No adjustment for multiple testing was applied. Data were analyzed using SPSS statistics 22.

## Results

We included 1013 consecutive patients with acute ischemic stroke during the outlined enrolment period. Mean age was 68.1 ± 13.4 years, 474 patients (46.8 %) were female and 243 patients (24.0 %) had suffered a stroke prior to the index stroke. Median NIHSS of all patients was 4 (IQR 2 – 10) on admission. Further baseline characteristics (co-morbidities, disability on admission, antithrombotic medication before admission) are shown in Table 1. During the in-hospital stay 37 (3.7 %) stroke patients died (median: 5.0 days after admission (IQR 2.0 - 7.5). Furthermore, 213 TIA patients were identified. Mean age of these patients was 68.3 ± 12.6 years, 108 patients (50.7 %) were female and 38 patients (17.8 %) had a stroke prior. None of these patients died in-hospital.

**Table 1:** Baseline characteristics of all 1013 patients with ischemic stroke, and of those with (*n* = 72) or without CIAD (*n* = 941), respectively

	All stroke patients *n* = 1013	Stroke patients without CIAD *n* = 941	Stroke patients with CIAD *n* = 72	p*
Age; median (IQR)	69.0 (61.0–78.0)	69.0 (60.0–78.0)	73.0 (65.3–78.8)	**0.042**
Female sex; % (n)	46.8 (474)	47.0 (442)	44.4 (32)	0.679
Comorbidities; % (n)				
Previous stroke or TIA	24.0 (243)	24.0 (226)	23.6 (17)	0.938
Atrial fibrillation	26.5 (268)	26.0 (245)	31.9 (23)	0.273
Diabetes	28.0 (284)	27.5 (259)	34.7 (25)	0.190
Hypertension	71.8 (727)	71.6 (674)	73.6 (53)	0.718
Coronary artery disease	21.6 (219)	21.0 (198)	29.2 (21)	0.106
Peripheral artery disease	6.0 (61)	5.7 (54)	9.7 (7)	0.171
Chronic heart failure	16.6 (168)	16.5 (155)	18.1 (13)	0.728
Mechanical heart valve	0.4 (4)	0.4 (4)	0.0 (0)	0.579
NIHSS^*†*^ score; % (n)				0.169
0 – 10 points	75.9 (769)	76.3 (718)	70.8 (51)	
≥ 11 points	24.1 (244)	23.7 (223)	29.2 (21)	
Modified Rankin Scale score; % (n)				0.080
0–1	16.9 (171)	17.4 (164)	9.7 (7)	
2–3	47.5 (481)	47.5 (447)	47.2 (34)	
4–5	35.6 (361)	35.1 (330)	43.1 (31)	
Antithrombotic medication prior to admission; % (n)				0.370
None	66.4 (672)	65.9 (619)	73.6 (53)	
Antiplatelets	28.8 (291)	29.5 (277)	19.4 (14)	
Vitamin K antagonist	4.0 (40)	3.7 (35)	6.9 (5)	
Vitamin K antagonist & antiplatelet	0.3 (3)	0.3 (3)	0.0 (0)	
Heparin, full-dose	0.5 (5)	0.5 (5)	0.0 (0)	
Heparin, full-dose & antiplatelet	0.1 (1)	0.1 (1)	0.0 (0)	
Thrombolysis with rt-PA; % (n)	7.8 (79)	7.7 (72)	9.7 (7)	0.351
In-hospital mortality; % (n)	3.7 (37)	3.6 (34)	4.2 (0)	0.742

*p values CIAD patients vs. *patients without CIAD; based on Fishers’ exact test, linear trend test or exact Mann–Whitney test, respectively;* ^*†*^ *National Institutes of Health Stroke Scale.* Bold indicates significant results

### Stroke patients with or without CIAD

Overall, 72 (7.1 %) of all stroke patients in this cohort had a medical history (*n* = 57) or an in-hospital diagnosis of CIAD (*n* = 16). No significant differences were observed between stroke patients with and without CIAD regarding gender, stroke type, main co-morbidities, prior medication or in-hospital mortality (Table 1). However, stroke patients with CIAD were slightly older (67.9 ± 13.5 vs. 71.0 ± 11.0, respectively; *p* = 0.042) and had a higher mRS on admission (median mRS 3 (IQR 2–5) vs. 3 (IQR 2–4); *p* = 0.047). Median NIHSS on admission of all stroke patients with or without CIAD was 5.0 (IQR 3.0 – 11.8) or 4.0 (IQR 2.0 – 10.0), respectively (*p* = 0.053). The prevalence of CIAD was similar in patients with TIA (7.4 %).

### Predictors for ischemic stroke severity on admission

On hospital admission, 24.1 % (*n* = 244) of all patients with ischemic stroke had a NIHSS ≥ 11 indicating severe stroke. In bivariate analysis, older age, female sex, co-existing atrial fibrillation, heart failure as well as coronary artery disease were associated with severe stroke (Table 2), while there was no impact of CIAD (OR 1.33 [95 % CI 0.78-2.25; *p* = 0.30). In multivariable analysis, age (OR 1.17 [95 % CI 1.03-1.37] per decade), a history of atrial fibrillation (OR 3.43 [95 % CI 2.47-4.78]) and coronary artery disease (OR 1.51 [1.07-2.14]) were independently associated with stroke severity on admission.

**Table 2:** Characteristics associated with stroke severity on hospital admission in 1013 patients with acute ischemic stroke (bivariate and multivariable analyses)

	Bivariate analyses			Multivariable analysis	
	Mild to moderate stroke (NIHSS < 11) *n* = 769	Severe stroke (NIHSS ≥ 11) *n* = 244	*p* value	Probability severe stroke OR (95 % CI)	*p* value
Age in years; median (IQR)	68.0 (59.5–77.0)	74.0 (65.0–81.0)	**<0.001**	1.17 (1.03–1.37)^*^	**0.018**
Female sex; % (n)	44.6 (343)	53.7 (131)	**0.013**	1.16 (0.84–1.59)	0.372
Comorbidities; % (n)					
Previous stroke or TIA	24.3 (187)	23.0 (56)	0.663		
Atrial fibrillation	19.1 (147)	49.6 (121)	**<0.001**	3.43 (2.47–4.78)	**<0.001**
Diabetes	27.8 (214)	28.7 (70)	0.794		
Hypertension	71.9 (553)	71.3 (174)	0.856		
Coronary artery disease	18.9 (145)	30.3 (74)	**<0.001**	1.51 (1.07–2.14)	**0.020**
Peripheral artery disease	6.0 (46)	6.1 (15)	0.924		
Chronic heart failure	13.7 (105)	25.8 (63)	**<0.001**	1.33 (0.89–1.97)	0.161
Chronic inflammatory airway disease	6.6 (51)	8.6 (21)	0.296		
Antithrombotic medication prior to admission; % (*n*)			0.631		
None	66.8 (514)	65.0 (158)			
Antiplatelets	28.0 (215)	31.3 (76)			
Anticoagulation INR < 2	2.6 (20)	1.6 (4)			
Anticoagulation INR ≥ 2^*†*^	2.6 (20)	2.1 (5)			

^*^OR are related to age in decades; ^†^or full-dose heparin (n = 5). Bold indicates significant results

When all patients with TIA (*n* = 213) were included in the multivariable analysis, age (OR 1.14 [95 % CI 1.01-1.30] per decade), a history of atrial fibrillation (OR 3.62 [95 % CI 2.62-5.00]) or chronic heart failure (OR 1.47 [CI 95 % 1.01-2.15]) were associated with higher stroke severity, while coronary artery disease (OR 1.40 [CI 95 % 0.99-1.98]) was not. Again, there was no significant association of CIAD (OR 1.31 [CI 95 % 0.78-2.18]; *p* = 0.31) with stroke severity on admission in this cohort of 1226 patients with ischemic stroke or TIA.

### Predictors for death in stroke patients

Follow-up data for 892 stroke patients (88.1 %) and 180 TIA patients (84.5 %) were available. Overall, 383 of 892 stroke patients (42.3 %) died during follow-up (median follow up: 80.0 months, IQR 32.0-85.0 months). Median survival time of patients who died was 25 months (IQR 3–52). There was no difference regarding pneumonia- or infection-associated death during the in hospital stay in patients with or without CIAD, respectively.

**Fig. 1:**
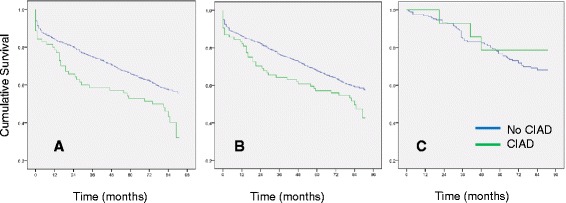
Kaplan-Meier estimates of cumulative survival after ischemic stroke by co-existing CIAD. Kaplan-Meier estimates of cumulative survival after ischemic stroke stratified by co-existing chronic inflammatory airway disease (CIAD) in **a** 892 patients with ischemic stroke (*p* = 0.009); **b** 892 patients with ischemic stroke and 179 patients with TIA (*p* = 0.036); **c** 179 patients with TIA (*p* = 0.469)

According to log-rank-test, co-existing CIAD was associated with death during long-term follow-up in patients with ischemic stroke (*p* = 0.009) as well as in patients with ischemic stroke or TIA (*n* = 1071, *p* = 0.036) (Fig. 1a and b) but not in TIA patients (*n* = 179; *p* = 0.469) (Fig. 1c). In multivariable analysis, age (HR 1.70 [95 % CI 1.53-1.87] per decade), peripheral artery disease (HR 1.91 [95 % CI 1.35-2.71]), and stroke severity on admission (NIHSS per point; HR 1.08 [95 % CI 1.08-1.10]) as well as CIAD (HR 1.43 [95 % CI 1.02-2.00) were significantly associated with death in long-term follow-up (Table 3). After including all patients with TIA and available follow-up in the analysis; age (HR 1.69 [95 % CI 1.54-1.85] per decade), coronary artery disease (HR 1.30 [95 % CI 1.04-1.62]), peripheral artery disease (HR 1.79 [95 % CI 1.28-2.49]), and stroke severity on admission (NIHSS per point HR 1.08 [95 % CI 1.06-1.10]) were associated with long-term mortality. There was a non-significant trend for lower long-term survival in stroke or TIA patients with CIAD according to multivariable analysis (HR 1.32 [95 % CI 0.96-1.81]; *p* = 0.093).

**Table 3:** Characteristics associated with long-term mortality in patients with acute ischemic stroke (*n* = 892). Median follow-up was 80 months (IQR 32–85)

	Bivariate analysis			Multivariable analysis	
	Survival *n* = 509	Death *n* = 383	*p* value	Probability death HR (95 % CI)	*p* value
Age in years; median (IQR)	65.0 (56.5–73.0)	75.0 (68.0–83.0)	**<0.001**	**1.70 (1.53–1.87)** ^*^	**<0.001**
Female sex; % (n)	45.0 (229)	51.2 (196)	0.065		
Comorbidities; % (n)					
Previous stroke or TIA	20.6 (105)	30.0 (115)	**0.002**	1.16 (0.92–1.46)	0.210
Atrial fibrillation	21.8 (111)	41.0 (157)	**<0.001**	1.08 (0.85–1.37)	0.523
Diabetes	23.0 (117)	36.0 (138)	**<0.001**	1.19 (0.96–1.48)	0.108
Hypertension	70.1 (357)	76.5 (293)	0.079		
Coronary artery disease	16.1 (82)	30.0 (115)	**<0.001**	1.26 (0.99–1.60)	0.053
Peripheral artery disease	3.5 (18)	10.2 (39)	**<0.001**	**1.91 (1.35-2.71)**	**<0.001**
Chronic heart failure	11.8 (60)	25.3 (97)	**<0.001**	1.13 (0.87–1.45)	0.361
Chronic inflammatory airway disease	5.9 (30)	10.4 (40)	**0.010**	**1.43 (1.02–2.00)**	**0.036**
Antithrombotic medication prior to admission; % (n)					
None	70.1 (357)	59.4 (227)			
Antiplatelets	24.0 (122)	36.1 (138)	**<0.001**	1.18 (0.94–1.48)	0.164
Anticoagulation INR < 2	2.8 (14)	2.4 (9)	0.786	0.94 (0.47–1.86)	0.858
Anticoagulation INR ≥ 2^*†*^	3.1 (16)	2.1 (8)	0.512	0.59 (0.29–1.12)	0.153
NIHSS^$^ on admission (per point); median (IQR)	3.0 (1–7)	7.0 (3–13)	**<0.001**	**1.08 (1.06–1.10)**	**<0.001**
Thrombolysis; % (n)	5.9 (30)	8.1 (31)	**0.034**		0.922

^*^ *OR are related to age in decades;* ^*†*^ *or full-dose heparin (n = 5);* ^$^ *National Institutes of Health Stroke Scale*. Bold indicates significant results

While age, stroke severity on admission as well as co-existing peripheral artery disease were significantly associated with mortality during short- (30 or 90 days after stroke onset; Table 4) and long-term follow-up (Table 3), co-existing CIAD was significantly associated with mortality only in the long term follow-up.

**Table 4:** Multivariable Cox-regression analysis of characteristics associated with mortality in patients with acute ischemic stroke during short-term follow-up (n = 892). Data are expressed as hazard ratios (95 % CI). All significant characteristics according to bivariate analyses (as similarly demonstrated in Table 3) were included. Analyses were adjusted for previous stroke or TIA, atrial fibrillation, diabetes, chronic heart failure, coronary artery disease, antithrombotic medication prior to admission, and thrombolysis)

	HR (95 % CI)	
	30 days	90 days
Age per decade	1.73 (1.39–2.17)	1.89 (1.54–2.31)
Peripheral artery disease	3.27 (1.67–6.42)	3.73 (2.08–6.70)
Chronic inflammatory airway disease	1.51 (0.76–3.01)	1.47 (0.77–2.82)
NIHSS^*^ on admission (per point)	1.22 (1.18–1.26)	1.19 (1.16–1.23)

^*^National Institutes of Health Stroke Scale score

## Discussion

This study demonstrated that co-existing CIAD is a frequent co-morbidity in patients with acute ischemic stroke. The main finding of this study is that CIAD was not significantly associated with stroke severity at hospital admission or with short-term outcome in stroke patients. In the long-term course after ischemic stroke, however, CIAD could be identified as a potential independent risk factor for mortality, extending the current knowledge on the role of CIAD as relevant co-morbidity after ischemic stroke (see Table 3). By contrast, other cardiovascular risk factors such as age, peripheral artery disease and stroke severity on hospital admission were confirmed as significant risk factors for short-term as well as long-term mortality [22–24] or risk factors for more severe stroke, respectively (Table 4) [25]. Interestingly, there was no significant impact of CIAD on outcome in patients with TIA but the number of TIA patients with CIAD was too low to draw final conclusions. Further studies are needed to validate the impact of CIAD on mortality after ischemic stroke.

CIAD and ischemic stroke are both primarily affecting the elderly and have a variety of risk factors in common. Therefore, a relevant proportion of patients suffer from both diseases. The observed CIAD prevalence of 7 % in stroke patients is higher compared to a Swedish cohort study (3.3 %), and higher compared to the general population (0.8 - 3.6 %) [8, 26].

According to current guidelines, CIAD is not considered a relevant co-morbidity to be addressed for primary or secondary stroke prevention [27, 28]. Our data on the long-term course after stroke underline that a relevant interaction between CIAD and stroke exists, as reported previously [4, 8, 14]. Notably, this interaction was not observed in the acute and subacute phase but emerged only in the prolonged course of the disease. It may be concluded that the CIAD related pathophysiologic signals are subtle and hence may not have a relevant impact on the acute processes after stroke. In the chronic course after stroke, however, the cumulative impact of the comorbidity adds to the overall pathophysiologic burden and results in the observed increase in mortality.

It may by hypothesized that the observed increase in long-term mortality in these patients is not merely relative to the CIAD itself as isolated condition but rather results from an interaction between the two diseases. Further studies are needed to uncover the pathophysiologic links and pathways underlying this interaction between cerebrovascular disease and systemic inflammation. This interaction between both co-morbidities seems highly relevant for the clinical evaluation of these patients [7]. Even more, as CIAD is a systemic inflammatory – yet treatable – disease and may therefore represent an additional risk factor for ischemic stroke [8, 11] and stroke-related morbidity. Hence, diagnosing and treating CIAD appears especially relevant in stroke patients. Prospective clinical studies (especially in patients with prior stroke) are needed to confirm whether optimal treatment of CIAD might reduce the burden of (recurrent) stroke, as suggested for major adverse cardiovascular events in the clinical database of placebo-controlled roflumilast chronic obstructive pulmonary disease trials [29] or recurrent stroke in montelukast-treated patients in a Swedish population-based cohort study [30].

Furthermore, our findings may reflect the importance of CIAD-related mortality. During the natural history of CIAD, patients initially die of cardiovascular causes or cancer but with advanced CIAD, respiratory failure and lung related mortality becomes more important [12, 16, 31, 32].

There are several limitations of the study that should be addressed: First, our study is a post hoc analysis that suffers from typical limitations of such studies. Several variables of interest were not recorded and were, therefore, not accessible for analysis. We do, for example, not have the information on the prevalence of obstructive sleep apnea. Furthermore, this study does not allow stratifying for symptomatic severity of CIAD - making it impossible to do a dose–response analysis -, subsequent functional impairment or medication schemes. Notably, unawareness of chronic diseases immediately after acute ischemic stroke may have resulted in underreporting of CIAD to a certain extent [33], thus creating a potential selection bias.

## Conclusion

In contrast to other cardiovascular risk factors, CIAD did not significantly interfere with severity of acute ischemic stroke on hospital admission and with short-term mortality after stroke. Compared to stroke patients without CIAD, co-existing CIAD increased the hazard of dying over longer term, suggesting the relevance of CIAD as a risk factor for patients after ischemic stroke. This finding underscores the need to identify and manage CIAD in those patients with acute ischemic stroke. It remains to be determined whether the incidence of recurrent stroke in CIAD patients is increased. This should be investigated in future studies.

## Abbreviations

AF: Atrial fibrillation
CHF: Chronic heart failure
CIAD: Chronic inflammatory airway disease
INR: International normalized ratio
mRS: Modified Rankin Scale
SD: Standard deviation
TIA: Transient ischemic attack
